# Measurement and analysis of equity in health: a case study conducted in Zhejiang Province, China

**DOI:** 10.1186/s12939-018-0746-8

**Published:** 2018-03-22

**Authors:** Xueshan Sun, Hao Zhang, Xiaoqian Hu, Shuyan Gu, Xuemei Zhen, Yuxuan Gu, Minzhuo Huang, Jingming Wei, Hengjin Dong

**Affiliations:** 1 Center for Health Policy Studies, School of Public Health, Zhejiang University School of Medicine, Hangzhou, China; 2 Hangzhou Normal University, Hangzhou, China

**Keywords:** Measurement, Equity in health, Health service utilization, Medical insurance

## Abstract

**Background:**

Equity is the core of primary care. The issue of equity in health has become urgent, and China has attached increasing attention to it. With rapid economic development and great changes in medical insurance policy, the pattern of equity in health has changed tremendously. The reform of healthcare in Zhejiang Province is at the forefront in China, and studies on Zhejiang Province are of great significance to the entire country. This paper aimed to measure health equity from the perspectives of health needs and health-seeking behavior and to provide suggestions for the next policy formulations, with respect to timeliness.

**Methods:**

The investigator’s household survey was conducted in August 2016. A sample of 1000 households, which included2807 individuals in Zhejiang, China, was obtained with the multi-stage stratified cluster sampling method. Descriptive analysis and chi-square tests were adopted in the analysis. The value of the concentration index was used to measure the equity.

**Results:**

This study found that the poor have more urgent health needs and poorer health situations than the rich. Through studies on health-seeking behavior, the utilization of outpatient services was almost equitable, while the utilization of hospitalization showed a pro-rich inequity (i.e., the rich use more services). Individuals with employer-based medical insurance used more outpatient services than those with rural and urban medical insurance. More people in the poorer income groups did not use inpatient services due to financial difficulties.

**Conclusions:**

Absolute medical prices and medical insurance may explain the equity in the utilization of outpatient services and the inequity in the utilization of hospitalization. In view of the pro-rich inequity of hospitalization, more financial protection should be provided for the poor.

## Background

In the past few decades, the issue of equity in health has aroused great attention. The world report noted that equity is the core of primary healthcare [1]. Equity usually refers to a relative concept of comparison between one person and another in a given situation [2]. In the field of health, equity can be interpreted to three parts, according to Whitehead: equal access to available care for equal need, equal utilization for equal need, and equal quality of care for all [3]. The connotation of equity in health consists of horizontal and vertical equity [4]. Horizontal equity implies that individuals with equal health needs should receive the same health services [5], and vertical equity represents unequal treatment of individuals in unequal levels of health needs [4, 6], individuals who have higher health needs should be given priority.

Equity in health has evolved in China. Before 1978, partly due to the wide coverage of medical insurance and the lack of significant income disparities, people could receive almost the same health services at an affordable cost, and inequities in health service utilization were not an urgent issue. After 1978, China started the process of economic reform, medical insurance coverage was shrunk [7], and the gap between the rich and the poor became wider. People had to pay for high medical expenses, while the poor could not support themselves due to financial difficulty. Inequalities in health were increasing [7]. From these changes, it can be concluded that income and medical insurance may play role in health equity. In 2000, China ranked 188^th^for health performance in a report of the WHO, which aroused much attention both nationally and globally. China has made great efforts to improve equities in health. The Central Committee of the Communist Party of China and the State Council issued a brand-new ‘Healthy China 2030’ in 2016 [8], which proposed the following: “Decrease the gaps of basic health services between individuals from urban and rural areas, different regions and different backgrounds…achieve national health coverage and promote equity…achieve equity in basic health services for urban and rural residents.”

China has an expansive amount of land with great differences in geographical conditions, resource situations and culture. Income disparities have been especially significant in China between different provinces and within provinces. The poor usually receive limited health services for their lack of money and weak health consciousness, while the rich can enjoy excessive and even unnecessary health services for their financial advantages, which suggest that we should pay more attention to whether the poor have access to the health services they need from both the horizontal and vertical perspective. One study showed that when dividing people into five groups according to their income, the annual hospitalization rates of the richest group is 14.3% higher than that of the lowest income group [4].However, a case study was also conducted in China showing that household income had no significant relationship with equity in health [9].The relationship between income disparities and equity in health will be further measured in this study.

China became entrenched in a system of employer-based medical insurance in 1998 [10], after which China started a new system of rural cooperative medical insurance and a medical assistance system [11]. In 2007, China provided medical insurance for urban residents [11]. In recent years, many provinces in China, including Zhejiang Province, have merged three medical insurance systems into two: employer-based medical insurance and rural and urban medical insurance. Previous studies have shown that medical insurance has played an important role in health equity. Some studies have shown that medical insurance has had a negative influence on the equity of outpatient services [4, 12, 13] and a positive influence on the equity of inpatient services [4, 12]. Other studies have shown the people with new rural cooperative medical insurance achieved equity in outpatient services, despite slight inequity in inpatient services [14, 15].

With rapid economic development and changes in medical insurance policy, the pattern of equity in health has changed tremendously in recent years. However, there remains a lack of targeted and practical suggestions based on data for improving health equity in a timely manner in China. Using Zhejiang Province as an example, this paper is aimed to measure equity in health; analyze the relationships among income disparities, medical insurance and equity in health; and provide more targeted and practical suggestions. The results and conclusions in this paper can be used in the formulation of policy.

## Methods

### Study design and sampling

The study was conducted in Zhejiang Province for its representative socioeconomic characteristics and frontier position in health care reform. Zhejiang is located in the South of China and had a GDP per capita of ¥83,538 ($12,576.67^1^) in 2016 [16, 17]; it is an economically developed city in China. Meanwhile, Zhejiang steadily carried out the pilot reform of the “three-in-one” hierarchical medical model, adopted an enhanced hierarchical medical insurance system and promoted the contracted service of responsible doctors, placing Zhejiang at the forefront of health care reform in China [18].

Two counties were chosen: Jiashan County and Jinyun County. The selection was based on two criteria: 1) one county represented a more developed area, and the other represented a less developed area (Jiashan County was the more developed area and Jinyun County was the less developed area, from the perspective of GDP); and 2) the governments of the two counties were able to support our project.

The study followed the principles of economy and effectiveness and adopted a multi-stage stratified cluster random sampling method. In each county,10 neighborhood committees of one street were randomly selected (towns), or 10 administrative villages were randomly selected in each township (rural areas). Finally, households in each neighborhood (towns) or administrative village (rural areas) were sampled using a random cluster sampling method. Since the proportions of the household-registered population and the floating population were different, the number of households was allocated according to the proportions of the household-registered population and the floating population. The sample size for this survey was 1000 households, which included 2899 individuals. A total of 96.8% individuals completed the survey, and we obtained 2807 valid questionnaires.

### Data collection

Data collection was carried out in August2016 by researchers from Zhejiang University and local health departments. A uniform standard questionnaire was used in all household surveys, and the questions related to this paper included the following: basic demographic and sociological characteristics (age, sex, income, marriage, education and occupation), disease status (prevalence of chronic disease in the last six months and the onset of sickness in the last two weeks), and utilization of health services (hospital visit status in the last two weeks and hospitalization in the last year).

We recruited one liaison and several researchers in local health-related departments to assist us in conducting the household survey. They were familiar with local conditions and possessed sufficient health-related knowledge. After standardized training, researchers organized the household surveys, and the male liaisons were responsible for communicating and supervising. Professors and students from Zhejiang University were responsible for checking the accuracy and completeness of the questionnaires. The researchers were asked to make corrections when the questionnaires were not qualified.

### Data analysis

Prior to statistical analysis, all questionnaires were exclusively coded and uploaded into a computerized database using EpiData 3.1. After upload, the data were analyzed with SPSS 21.0. Descriptive analysis was used to analyze the demographic and sociological characteristics of the sample of individuals. Chi-square tests were adopted to analyze differences in health service utilization and health needs among the different income groups.

We adopted household per-capita income as a standard by which to divide the individuals into five groups: lowest, low, middle, high, highest. Five income groups were successively encoded as “I, II, III, IV, V”, and each group had almost1/5 of the total number of individuals. For urban individuals, their household income was disposable income, and for rural individuals, their household income was net income during the entire year of 2015 (Table 1).

**Table 1:** Income levels by quintile

Income groups	Group code	Proportion (%)	Per capita per year ($)	Income share (%)	Cumulative income share (%)
Lowest	I (*n* = 628)	22.37	979.08	5.48	5.48
low	II (*n* = 537)	19.13	2205.65	10.54	16.02
Middle	III (*n* = 541)	19.29	3329.97	16.04	32.06
High	IV (*n* = 509)	18.13	4774.14	21.64	53.70
Highest	V (*n* = 592)	21.09	8784.34	46.30	100.00

The criterion used to judge inequities in healthcare was the value of the concentration index (CI); individuals were ranked not by their health status but by their socioeconomic status, beginning with the most disadvantaged and ending with the most advantaged [19]. In this study, people were ranked by their per-capita household income. There are no income-related inequities if the value of the CI is zero, and when the CI is positive (negative), it expresses that there are pro-rich (pro-poor) inequities in health [20]. Geometric methods were used in this study to obtain the CI. The formula is as follows [21–23]:

$$ \mathrm{CI}=1-{\sum}_{i=0}^{n-1}\left({Y}_i+{Y}_{i+1}\right)\left({X}_{i+1}-{X}_i\right)\left(\mathrm{X}0=\mathrm{Y}0=0\right) $$

In this formula, CI is concentration index. X_i_ represents the cumulative percentage of population in level i, and Y_i_ represents the cumulative percentage of health indicators in level i [21].

### Variable settings

The indicators “prevalence of illness in the last 2 weeks”, “prevalence of chronic disease in the last 6 months” and “self-perceived health status” were adopted to analyze the health needs of the individuals. The “prevalence of illness in the last 2 weeks” equals the number of people who reported to be sick in the last 2 weeks divided by the number of individuals in the entire sample [24]. The “prevalence of chronic disease in the last 6 months” equals the number of people who had chronic disease in the last 6 months divided by the number of individuals in the sample [24]; the “self-perceived health status” equals the number of people who felt unhealthy in the last 1 year divided by the number of individuals in the sample.

The “2-week prevalence of absence rate” was adopted to analyze the utilization of outpatient services. The “2-week prevalence of absence rate” equals the number of people who reported illness but did not utilize outpatient services, divided by the number of people who reported illness in the last 2 weeks. The “non-hospitalization rate” was used to analyze the utilization of inpatient services. The “non-hospitalization rate” equals the number of people who should have been hospitalized but were not, divided by the number of people who should have been hospitalized.

## Results

### Sociodemographic characteristics

A total of 2899 individuals were enrolled, and 2807 valid individuals were included in this study. Among them, 1419 individuals were from Jiashan County, and 1388 individuals were from Jinyun County.

Among the 2807 individuals, 22.8% were aged over 60 years old, and 49.7% were male, which was slightly lower than the proportion of females. A total of 8.8% were illiterate, and 30.4% had a secondary school degree. In total, 57.7% were employed. Only 2.6% did not have insurance coverage, 65.4% had medical insurance for rural and urban residents, and 30.8% had employer-based medical insurance (Table 2).

**Table 2:** Sociodemographic characteristics

Variables	I (n(%))	II (n(%))	III (n(%))	IV (n(%))	VI (n(%))	Total (n(%))
Gender						
Female	302(48.1)	268(49.9)	272(50.3)	254(49.9)	298(50.3)	1394(49.7)
Male	326(51.9)	269(50.1)	269(49.7)	255(50.1)	294(49.7)	1413(50.3)
Age(years)						
0–10	49(7.8)	43(8.0)	33(6.1)	31(6.1)	43(7.3)	199(7.1)
11–20	55(8.8)	55(10.2)	46(8.5)	44(8.6)	43(7.3)	243(8.7)
21–30	65(10.4)	72(13.4)	68(12.6)	64(12.6)	60(10.1)	329(11.7)
31–40	61(9.7)	70(13.0)	68(12.6)	84(16.5)	110(18.6)	393(14.0)
41–50	118(18.8)	103(19.2)	105(19.4)	109(21.4)	112(18.9)	547(19.5)
51–60	99(15.8)	64(11.9)	79(14.6)	88(17.3)	125(21.1)	455(16.2)
Over 60	181(28.8)	130(24.2)	142(26.2)	89(17.5)	99(16.7)	641(22.8)
Education						
Illiteracy	77(12.3)	50(9.3)	48(8.9)	32(6.3)	41(6.9)	248(8.8)
Primary school	258(41.1)	169(31.5)	171(31.6)	121(23.8)	83(14.0)	802(28.6)
Secondary school	200(31.8)	184(34.3)	173(32.0)	167(32.8)	129(21.8)	853(30.4)
High school	39(6.2)	62(11.5)	49(9.1)	48(9.4)	73(12.3)	271(9.7)
Technical school	28(4.5)	27(5.0)	30(5.5)	35(6.9)	39(6.6)	159(5.7)
University/college	26(4.1)	45(8.4)	70(12.9)	106(20.8)	227(38.3)	474(16.9)
Employment						
Employed	327(52.1)	308(57.4)	307(56.7)	319(62.7)	359(60.6)	1620(57.7)
Retired	29(4.6)	70(13.0)	91(16.8)	78(15.3)	122(20.6)	390(13.9)
Student	85(13.5)	76(14.2)	71(13.1)	64(12.6)	74(12.5)	370(13.2)
Unemployed	187(29.8)	83(15.5)	72(13.3)	48(9.4)	37(6.3)	427(15.2)
Insurance						
None	11(1.8)	11(2.0)	13(2.4)	18(3.5)	20(3.4)	73(2.6)
Employer-based	53(8.4)	119(22.2)	152(28.1)	189(37.1)	351(59.3)	864(30.8)
Urban and rural	561(89.3)	404(75.2)	358(66.2)	298(58.5)	215(36.3)	1836(65.4)
Commercial	3(0.5)	3(0.6)	11(2.0)	4(0.8)	5(0.8)	26(0.9)
Other	0(0)	0(0)	7(1.3)	0(0)	1(0.2)	8(0.3)

### Health needs

It can be found that individuals in groups with lower income were in poorer health, and their health needs were more urgent As shown in Table 3, a significant difference can be found for the indicator “prevalence of illness in the last 2 weeks” among five income groups (*P* < 0.05). The prevalence of illness in the last 2 weeks of the lowest income group was 16.1%, and the number of individuals who reported illness in this group was 101, accounting for 30.8% of the total number of individuals who reported illness in the last 2 weeks. The CI of prevalence of illness in the last 2 weeks was negative (CI = − 0.067), indicating that the inequality in the prevalence of illness in the last 2 weeks and the burden of illness was mainly concentrated among individuals in groups with lower income.

**Table 3:** health needs and health service utilization

Indicators	I (%)	II (%)	III (%)	IV (%)	VI (%)	P	CI
Prevalence of illness in the last 2 weeks	16.1 (101/628)	9.5 (51/537)	10.9 (59/541)	10.4 (53/509)	10.8 (64/592)	0.003	−0.067
Prevalence of chronic disease in the last 6 months	15.3 (96/628)	10.6 (57/537)	12.9 (70/541)	10.4 (53/509)	9.6 (57/592)	0.015	−0.079
Self-perceived unhealthy rate	7.2 (45/628)	4.5 (24/537)	2.6 (14/541)	1.0 (5/509)	2.2 (13/592)	0.000	−0.309
2-week prevalence of absence rate	38.6 (39/101)	54.9 (28/51)	52.5 (31/59)	64.2 (34/53)	35.9 (23/64)	0.007	0.003
Non-hospitalization rate	36.4 (24/66)	47.1 (24/51)	35.7 (25/70)	11.4 (4/35)	3.2 (1/31)	0.000	−0.305

A significant difference can be found for the indicator “prevalence of chronic disease in the last 6 months” among five income groups (*P* < 0.05). The prevalence of chronic diseases in the last 6 months in the lowest income group was 15.5%, while the prevalence of chronic diseases in the last 6 months in the highest income group was 9.6%. The number of individuals who reported chronic diseases in the lowest income group was 96, accounting for 28.8% of the total number of individuals who reported chronic diseases in the last 6 months. The CI of the prevalence of chronic disease in the last 6 months was − 0.079, showing the burden of chronic disease was concentrated among individuals in groups with lower income.

A total of 7.2% of individuals in the lowest income group felt unhealthy, while the rate of the high-income group was 1.0%. The number of individuals who felt unhealthy in the lowest income group accounted for 44.6% of the total number individuals who felt unhealthy. The CI of the self-perceived health status was lower than zero (CI = − 0.309). Thus, the burden of the self-perceived health status was mainly concentrated among the lower income groups, especially the lowest income group.

On the whole, taking the abovementioned three indicators into consideration, individuals in groups with lower income were in poorer health than individuals in groups with higher income, and their health needs were more urgent.

### Health services utilization

It can be found that equity in outpatient service utilization with inequity in inpatient service utilization. Utilization of outpatient services among individuals in groups with lower income was not lower than that among individuals in groups with higher income. The CI of the “2-week prevalence of absent rate” was 0.003, which was slightly higher than zero, indicating that individuals in groups with lower income used slightly more outpatient services than individuals in groups with higher income. This finding can almost reflect equity in the indicator “2-week prevalence of absent rate” between the rich and the poor. Despite the low purchasing power of poor people, there was still no obvious inequity in outpatient service utilization.

Although the utilization of outpatient services essentially showed equity, there remained significant inequality in inpatient service utilization. The non-hospitalization rate of individuals from the lowest income group was 36.4%, while that of individuals from the highest income group was only 3.2%; thus, the gap was large. Of 78 individuals who reported non-hospitalization behavior, there were only 5 individuals in the high or the highest income groups. The CI of the non-hospitalization rate was lower than zero (CI = − 0.305), indicating a significant inequity in inpatient service utilization (Table 3).

### Reasons for non-hospitalization

According to physicians’ diagnoses, there were 253 individuals who should have been hospitalized, 78 of whom were not. It was found that 26 individuals were not hospitalized due to financial difficulties, 21 of whom were from the lowest or the low-income groups. A total of 17 individuals thought it unnecessary, 10 of whom were from the lowest income group. In the highest income group, there was just one case of non-hospitalization. Non-hospitalization of the poor due to financial difficulties should be given more attention (Table 4).

**Table 4:** Reasons for non-hospitalization by income level

Groups	Unnecessary	Ineffective measure	Financial difficulties	Poor hospital service	No time	No enough bed	other
I	10	1	9	1	1	0	2
II	2	0	13	0	0	2	7
III	3	0	4	0	0	0	18
IV	1	0	0	0	0	0	3
VI	1	0	0	0	0	0	0
Total	17	1	26	1	1	2	30

### Equity of health services utilization by two types of medical insurance

It can be found that individuals with urban and rural medical insurance used fewer inpatient services. There were 864 individuals with employer-based insurance and 1836 individuals with urban and rural insurance, comprising 96.2% of the total number of participants in this study. Zhejiang Province has almost achieved universal medical coverage.

The benefits packages of the two types of medical insurance were different. It is necessary to explore the health service utilization of individuals with different medical insurance types. There was no significant difference in the indicator “2-week prevalence of absence rate” between the group with employer-based medical insurance and the group with urban and rural medical insurance (*P* > 0.05). There was a significant difference in the indicator “non-hospitalization rate” between the group with employer-based medical insurance and the group with urban and rural medical insurance (*P* < 0.05). Different individuals had almost equal utilization of outpatient services, while their utilization of inpatient services was not equal. Individuals with employer-based medical insurance use more inpatient services.

The results of the health service utilization for the two types of medical insurance were consistent with the CI of the health service utilization shown in Table 5.

**Table 5:** Health service utilization by two types ofmedical insurance

Indicators	Employer-based (% (n/N))	Urban and rural (% (n/N))	*P* value
2-week prevalence of absence rate	54.8 (57/104)	43.8 (96/219)	0.065
Non-hospitalization rate	3.3 (2/60)	34.7 (61/176)	0.000

## Discussions

The health needs of individuals in groups with lower income were more urgent than those of individuals in groups with higher income. In reality, there remains the barrier of purchasing power between need and utilization. Individuals in groups with lower income are usually faced with financial difficulties, and although they have more urgent health needs, their utilization of health services will still be lower than that of individuals in groups with higher income. However, this study indicates inequity in inpatient services and equity in outpatient services, which is consistent with the findings from Xing’s studies on health equity that were conducted in Zhejiang in 2015 [25].

Medical insurance may have played an important role in health equity. There are two major types of medical insurance in Zhejiang: employer-based medical insurance and urban and rural medical insurance. Theoretically speaking, due to different financing standard, the benefit packages of two types of medical insurance were different and individuals with urban and rural medical insurance couldn’t be able to enjoy medical resources equitably with individuals with employer-based medical insurance [26]. There remains a gap in the reimbursement between the two types of insurance. Individuals with urban and rural medical insurance use fewer hospitalization services than individuals with employer-based medical insurance, partly due to the relatively low reimbursement. Meanwhile, in this paper, 59.3% individuals of the highest income group and 8.4% individuals of the lowest income group took part in employer-based medical insurance. In other words, 59.3% individuals of the highest income group enjoyed higher reimbursement, while only 8.4% individuals of the lowest income group enjoyed higher reimbursement. The relatively low reimbursement of rural and urban medical insurance aggravated the economic burden of individuals in groups with lower income, increasing the inequity in hospitalization.

Absolute price may greatly affect the equity of health. The poor are more sensitive to price changes [27], and they are also sensitive to the price itself. Therefore, they are more sensitive to the absolute price. The absolute price of outpatients was relatively lower. In this paper, per-capita outpatient expenses were¥1391.57 ($211.27). After reimbursement, the expenses were¥833.57 ($126.56), which was far lower than the average income of group with the lowest income ¥6503($979.08). Even individuals in groups with lower income can obtain outpatient services at an affordable cost. There remained a small barrier between health needs and outpatient utilization for all individuals, which can explain the equity of outpatient service utilization. However, the absolute price of hospitalization was relatively higher. In this paper, per-capita hospitalization expenses were¥15,892.14 ($2411.92). After reimbursement, the expenses were ¥8664.12 ($1314.82), which exceeded the average income of individuals in group with the lowest income ¥6503($979.08). Although individuals in groups with lower income had more urgent health needs than the rich, they usually avoided hospitalization due to financial difficulties, which can explain the equity in inpatient service utilization.

Improvements in health equity should start with the formulation of policy. For equity of outpatient services, Zhejiang established “Outpatient Medical Expenses Coordination” during the period of “The Twelfth Five Year Plan”, decreasing the financial burden of outpatient expenses [28]. In 2012, Zhejiang issued reforms in the personal accounts of employer-based insurance and conducted the family mutual aid system [29]. Personal accounts can be used to pay the expenses of drugstores and outpatient services, both for the insured person and his or her family members. Both systems have improved the equity of outpatient services utilization [30] and should be improved upon in the future.

However, compared with the equity of outpatient services, the inequity of hospitalization needs to be given more attention, as shown by the pro-rich inequity in hospitalization. Individuals who had non-hospitalization behavior due to financial difficulties were vulnerable groups in society, and their health status was poorer. The behavior of non-hospitalization increases the burden of disease for both individuals and society. The absolute price and the reimbursement of medical insurance cannot be easily changed in a short period of time due to the rising health care costs and increasing pressure on the pool of medical insurance funds. To improve the inequity of hospitalization, the focus of policy reform should be placed on the stimulation of the use of hospitalization for the poor. First, Zhejiang has a standard medical assistance system for the poor, but the application procedure is a complicated and involved process. It is hoped that more vulnerable groups can be brought into the medical assistance system and that the application procedure can be simplified. In addition to the medical assistance system, Zhejiang also set up a temporary assistance system called “Red Cross Aid”, which can give poor people medical support when they suffer from serious illness.“Red Cross Aid” should be improved upon in the future as a supplemental medical support for the poor. Finally, in the next formulation of policy, if hospitalization could be included into the payment of individual accounts, this change could stimulate the utilization of inpatient services and, as a result, improve the inequity in hospitalization. The expansion of individual account payments can also accelerate the promotion of the overall medical insurance system, thus increasing equity in the reimbursement of hospitalization, regardless of the different socioeconomic characteristics of insured persons.

There are three limitations in this study. First, this study made good use of the quantitative data of Zhejiang. If qualitative data are available as a supplement, they would better explain the results of the quantitative data analysis. Second, we conducted the survey in Qinghai, and the data has not been collected. In the next step of this study, we will draw a comparison between Zhejiang and Qinghai to further discuss the differences between different regions. Finally, 2807 individuals were enrolled in this study, and it is believed that if more individuals were enrolled, the analysis results would be more convincing.

## Conclusions

Our findings show that the health needs of individuals in groups with lower income were more urgent than those of individuals in groups with higher income. Outpatient service utilization of different individuals from different income groups showed equity, while hospitalization utilization of different individuals from different income groups showed inequity; furthermore, hospitalization shows a pro-rich inequity. Medical insurance and absolute medical expenses may play a role in the equity or inequity in healthcare. The policies of “Outpatient Medical Expenses Coordination”, the family mutual aid system and “Red Cross Aid” should be improved upon to increase health equity. In addition, whether the payment of individual accounts should be expanded to hospitalization should be further discussed.

## Abbreviation

CI: concentration index

## Footnotes

1 According to the People’s Republic of China’s 2016 National Economic and Social Development Statistical Bulletin, the average RMB exchange rate in 2016 was 1Dollar for 6.6423Yuan.
